# On the use of cues to assess attention in dyslexia

**DOI:** 10.3389/fnhum.2014.00983

**Published:** 2014-12-05

**Authors:** Bernt C. Skottun

**Affiliations:** Oslo, Norway

**Keywords:** perception, cues, spatial attention, visual attention, dyslexia, reading, reading disability

Several authors have sought to use cues to assess visual spatial attention in connection with dyslexia (e.g., Brannan and Williams, 1987; Facoetti et al., 2000, 2003; Franceschini et al., 2012; Ruffino et al., 2014). The idea behind this practice is that the cue causes attention to be directed to its location so that sensitivity increases here. Studies have generally found that dyslexic readers benefit less from cues than do controls (Facoetti et al., 2000, 2003; Ruffino et al., 2014). These results have been interpreted as evidence for deficient spatial attention in dyslexic readers. However, it is not clear that these results could not have been these results could not have been caused by perceptual abnormalities.

This possibility can be exemplified by the study of Facoetti et al. (2000) in which the effects of cues upon response time was determined at short (136 and 238 ms) and long (504 and 1000 ms) Stimulus Onset Asynchronies (SOAs). It was found that dyslexic readers did not benefit from cues at the shorter SOAs but did so at the longer SOAs. This was interpreted to mean that dyslexic readers have problems in relation to orienting and focusing attention.

A number of investigators have reported deficient visual perception in dyslexic subjects. For instance, contrast sensitivity deficits of a variety of kinds have been reported (Skottun, 2000). And, there is evidence to suggest that these deficiencies are not the secondary effects of attentional deficits (Skottun and Skoyles, 2007). The cues used in cueing experiments are typically very brief. In the case of Facoetti et al. (2000) they were of 99 ms duration. It seems therefore relevant that Gross-Glenn et al. (1995) found reduced contrast sensitivity in dyslexic subjects to high spatial frequency stimuli presented for short durations (≤102 ms). If a brief stimulus were harder to process it would presumably take a longer time for the visual system to recover from it so as to be ready for the processing of a second stimulus. This could cause poorer performance at short SOAs but not at long ones. Consistent with this Di Lollo et al. (1983, p. 923) found that “the dyslexic visual system may take an unusually long period of time to recover from the aftereffects of neural activity evoked by an inducing stimulus.” Also the more general suggestion that dyslexia may be associated with a temporal deficiency seems relevant (Farmer and Klein, 1995).

Further, the spatio-temporal arrangement of the stimuli used by Facoetti et al. (2000) (with the cue presented slightly before and above the target), most likely, contained motion energy. It is well-established that dyslexic readers have deficiencies linked to motion perception (see Skottun and Skoyles, 2006, for references). Irrespective of whether or not motion was actually seen, dyslexic readers may have had abnormal responses to such stimuli.

Facoetti et al. (2000) speculate that the poorer performance on the part of the dyslexic group may be related to a magnocellular deficit. It is difficult to understand how such a deficit, were it to exist, would not have been in a position to interfere with the effectiveness of cues without the involvement of attention.

Based on purely perceptual considerations it is, therefore, possible that dyslexic readers may have difficulties responding, and may have responded more slowly, to the second of two stimuli presented in rapid succession.

Roach and Hogben (2004) tested sensitivity to flicker and global dot motion in addition to the effects of cues. They found essentially normal detection thresholds to a flickering (10 Hz) Gaussian blob (i.e., no support for a magnocellular deficit) and global-dot-motion thresholds for the dyslexic subjects fell “toward the higher end of the normal range.” (In a follow up study Roach and Hogben, 2007, found a significant impairment in global motion perception.) Roach and Hogben (2004) concluded that the observed spatial-cueing deficit was not merely a secondary consequence of sensory dysfunction. However, it is not clear that the sensitivity to a large flickering “blob” is an appropriate test for assessing the ability to accurately localize a cue.

More relevant is therefore the study of Roach and Hogben (2008) in which cues were matched in regard to localization accuracy. It was found that even with matched cues dyslexic readers benefited less from cues than did controls. However, the difference between dyslexic subjects and controls were larger in the non-matched than in the matched cue conditions. (In the unmatched condition the ratio of orientation discrimination thresholds was 2.66, i.e., 13.25 deg/4.99 deg, whereas in the matched condition the ratio was 1.89, i.e., 10.63 deg/5.63 deg. See also their Figure 7). This suggests an at least partial influence of perceptual factors upon the effect of cues in relation to dyslexia.

In conclusion, therefore, in the case of dyslexia it is possible that the lack of effects of cues may reflect, in part or fully, perceptual deficiencies. This possibility needs to be considered when interpreting the findings from cueing experiments involving dyslexic subjects.

## Conflict of interest statement

The author declares that the research was conducted in the absence of any commercial or financial relationships that could be construed as a potential conflict of interest.

## References

[B1] Brannan J. Williams M. (1987). Allocation of visual attention in good and poor readers. Percept. Psychophys. 41, 23–28. 10.3758/BF03208209 3822740

[B2] Di Lollo V. Hanson D. McIntyre J. S. (1983). Initial stages of visual information processing in dyslexia. J. Exp. Psychol. Hum. Percept. Perform. 9, 923–935. 10.1037/0096-1523.9.6.923 6227701

[B3] Facoetti A. Lorusso M. L. Paganoni P. Cattaneo C. Galli R. Umilta C.. (2003). Auditory and visual automatic attention deficits in developmental dyslexia. Brain Res. Cogn. Brain Res. 16, 185–191. 10.1016/S0926-6410(02)00270-7 12668226

[B4] Facoetti A. Paganoni P. Turatto M. Marzola V. Mascetti G. G. (2000). Visual-spatial attention in developmental dyslexia. Cortex 36, 109–123. 10.1016/S0010-9452(08)70840-2 10728901

[B5] Farmer M. Klein R. (1995). The evidence for a temporal processing deficit linked to dyslexia. A review. Psychon. Bull. Rev. 2, 460–493. 10.3758/BF03210983 24203785

[B6] Franceschini S. Gori S. Ruffino M. Pedrolli K. Facoetti A. (2012). A causal link between visual spatial attention and reading acquisition. Curr. Biol. 22, 814–819. 10.1016/j.cub.2012.03.013 22483940

[B7] Gross-Glenn K. Skottun B. C. Glenn W. Kushch A. Lingua R. Dunbar M.. (1995). Contrast sensitivity in dyslexia. Vis. Neurosci. 12, 153–163. 10.1017/S0952523800007380 7718496

[B8] Roach N. W. Hogben J. H. (2004). Attentional modulation of visual processing in adult dyslexia–A spatial-cuing deficit. Psychol. Sci. 15, 650–654. 10.1111/j.0956-7976.2004.00735.x 15447634

[B9] Roach N. W. Hogben J. H. (2007). Impaired filtering of behaviourally irrelevant visual information in dyslexia. Brain 130, 771–785. 10.1093/brain/awl353 17237361

[B10] Roach N. W. Hogben J. H. (2008). Spatial cueing deficits in dyslexia reflect generalised difficulties with attentional selection. Vision Res. 48, 193–207. 10.1016/j.visres.2007.11.001 18068752

[B11] Ruffino M. Gori S. Boccardi D. Molteno M. Facoetti A. (2014). Spatial and temporal attention in developmental dyslexia. Front. Hum. Neurosci. 8:331. 10.3389/fnhum.2014.00331 24904371 PMC4033052

[B12] Skottun B. C. (2000). The Magnocellular deficit theory of dyslexia: the evidence from contrast sensitivity. Vision Res. 40, 111–127. 10.1016/S0042-6989(99)00170-4 10768046

[B13] Skottun B. C. Skoyles J. (2007). Dyslexia: sensory deficits or inattention? Perception 36, 1084–1088. 10.1068/p5468 17844973

[B14] Skottun B. C. Skoyles J. R. (2006). Is coherent motion an appropriate test for magnocellular sensitivity? Brain Cogn. 61, 172–180. 10.1016/j.bandc.2005.12.004 16455172

